# Cyanolichen microbiome contains novel viruses that encode genes to promote microbial metabolism

**DOI:** 10.1038/s43705-021-00060-w

**Published:** 2021-10-15

**Authors:** Alise J. Ponsero, Bonnie L. Hurwitz, Nicolas Magain, Jolanta Miadlikowska, François Lutzoni, Jana M. U’Ren

**Affiliations:** 1 BIO5 Institute and Department of Biosystems Engineering, University of Arizona, Tucson, AZ 85721 USA; 2 Department of Medicine, University of Helsinki, Helsinki, Finland; 3 Department of Biology, Duke University, Durham, NC 27708 USA; 4 Evolution and Conservation Biology, InBioS, University of Liège, Liège, Belgium

**Keywords:** Symbiosis, Metagenomics, Microbiome

## Abstract

Lichen thalli are formed through the symbiotic association of a filamentous fungus and photosynthetic green alga and/or cyanobacterium. Recent studies have revealed lichens also host highly diverse communities of secondary fungal and bacterial symbionts, yet few studies have examined the viral component within these complex symbioses. Here, we describe viral biodiversity and functions in cyanolichens collected from across North America and Europe. As current machine-learning viral-detection tools are not trained on complex eukaryotic metagenomes, we first developed efficient methods to remove eukaryotic reads prior to viral detection and a custom pipeline to validate viral contigs predicted with three machine-learning methods. Our resulting high-quality viral data illustrate that every cyanolichen thallus contains diverse viruses that are distinct from viruses in other terrestrial ecosystems. In addition to cyanobacteria, predicted viral hosts include other lichen-associated bacterial lineages and algae, although a large fraction of viral contigs had no host prediction. Functional annotation of cyanolichen viral sequences predicts numerous viral-encoded auxiliary metabolic genes (AMGs) involved in amino acid, nucleotide, and carbohydrate metabolism, including AMGs for secondary metabolism (antibiotics and antimicrobials) and fatty acid biosynthesis. Overall, the diversity of cyanolichen AMGs suggests that viruses may alter microbial interactions within these complex symbiotic assemblages.

Lichens—defined as the symbiotic association between a filamentous fungus (mycobiont) and at least one photosynthetic organism (photobiont)—grow on a broad array of substrates in terrestrial, freshwater, and marine intertidal ecosystems from the poles to the tropics [1]. The vast majority of lichens contain green alga (Chlorophyta) as their main photobiont, but >1500 species of lichen-forming fungi have *Nostoc* cyanobacteria either as primary photobionts (forming bi-membered lichens) or as secondary photobionts (forming tri-membered lichens with a green alga as the primary photobiont) [2]. In exchange for photosynthate (and fixed nitrogen from *Nostoc* cyanobiont), the fungal partner provides the photobiont with carbon dioxide, inorganic ions, and protection from light [2]. More recently, molecular studies have shown that lichens also contain cryptic secondary bacterial and fungal symbionts [3–5], yet few studies to date have examined viruses that associate with these complex microbial communities (but see [6]).

Viruses infect all domains of life and are the most abundant biological entity on Earth [7]. Previous studies have shown that viruses that infect bacteria are evolutionarily tuned to their hosts in a given environment toward two main goals: (i) promotion of viral replication for lysis or (ii) co-existence within the host genome as a prophage [8]. To do so, viruses can encode host genes (i.e., auxiliary metabolic genes (AMGs; [9]) that promote viral replication through manipulation of the host’s metabolism. For example, marine cyanophages encode *psbA* to increase host photosynthesis and drive replication when host *psbA* is inhibited by high light [10]. Viral AMGs can provide important clues into host adaptation, metabolic bottlenecks, key ecosystem functions, and interactions among members of a microbial community [8].

Viral sequences in metagenomes can be detected using reference-based methods, but these methods often are hampered by the limited diversity of viral genomes in reference databases (see [11]). Thus, an emerging approach is to apply machine-learning (ML) algorithms that use composition-based pattern detection. ML models identify a set of features that signal a viral origin (e.g., relative synonymous codon usage, gene density, strand shifts, number of hits to the Prokaryotic Virus Orthologous Groups (pVOGs) database), thus generalizing the identification of all viral sequences and enabling better detection of novel viruses [11–13]. These new approaches provide exciting avenues for detecting novel viral sequences in metagenomes and are key to investigating complex microbial symbioses such as lichens.

Here, we explored viral biodiversity in 11 cyanolichen metagenomes (Supplementary Table S1) representing nine species of the genus *Peltigera* sampled from North America, Finland, Iceland, and Panama, and one species from the sister genus *Solorina* (Fig. 1a) [14]. We predicted viruses with three ML tools (MARVEL, Vibrant, VirSorter) [11–13]. However, as repetitive regions of eukaryotic genomes can be falsely identified as viral [15], we first tested the false positive rate for each ML tools using a mock lichen community (Supplementary Table S2; Supplementary Fig. S1) and then developed a pipeline to remove eukaryotic sequences prior to viral detection and apply a stringent cutoff to limit false positives (i.e., minimum of 10% of open reading frames (ORFs)/contig hit a previously identified viral protein) (Supplementary Fig. S2).

**Fig. 1:**
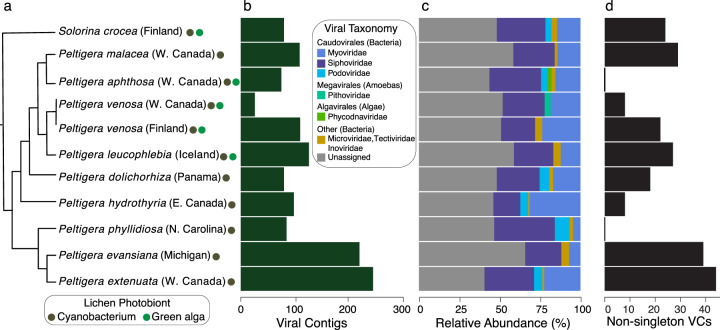
Cyanolichens harbor diverse viral communities that infect prokaryotic and eukaryotic hosts in lichen thalli. **a** Schematic tree illustrating the phylogenetic relationships among lichen mycobionts (Peltigerales) (see Supplementary Materials). Colored circles indicate the photobiont composition of each lichen (see legend). Names associated with two colored circles are tri-membered lichens, whereas names associated with one colored circle are bi-membered cyanolichens (see Supplementary Table S1 for photobiont details). **b** Bar graph of the number of cyanolichen viral contigs predicted per sample, which accounted for 0.6–3.4% of the total reads per sample. **c** Taxonomic classification of viral contigs identified 15 viral families that infect prokaryotic (*n* = 802 contigs) and eukaryotic hosts (*n* = 28 contigs), although 471 (36.2%) contigs could not be classified (Supplementary Table S3). **d** Distribution of non-singleton viral clusters (VCs) among samples. Among the 133 VCs, 50 were unique to a cyanolichen sample.

Every cyanolichen thallus contained putative viral sequences (range 27–254; Fig. 1b), although we observed no pattern between viral abundance and thallus type (bi- or tri-membered) or geographic location (Fig. 1b). In total, we predicted 1301 non-redundant viral contigs with high confidence across all metagenomes (Supplementary Fig. S3; Supplementary Table S3), including 116 predicted prophage sequences and 27 complete viral genomes that represent 8.9% and 2.0% of total contigs, respectively. The majority of viral contigs were classified as bacteriophages from the *Caudovirales* (61.2%) (Fig. 1c). Twenty-eight contigs also matched nine viral families that infect eukaryotes, including viruses of *Phycodnaviridae* that infect green algae [16] (Fig. 1c). Our taxonomic results are consistent with the observed bacterial communities in these samples (dominated by Proteobacteria and Cyanobacteria; Supplementary Fig. S4a), as well as our *in silico* predictions of viral hosts (Supplementary Fig. S4b). Additional eukaryotic viruses likely associate with algal photobionts of tri-membered lichens (see Fig. 1a) and fungal mycobionts in lichen thalli [6], but biases in both computational methods and databases towards phages may have limited their detection and classification (e.g., 36.2% of viruses were unclassified; Fig. 1c; Supplementary Table S3). Additionally, many algal and fungal viruses are dsRNA viruses that cannot be detected with metagenomic data [6, 17].

To assess the novelty of cyanolichen viruses, we compared our contigs to >400000 previously published terrestrial viral contigs and genomes. The majority of our contigs (*n* = 966) did not form viral clusters (VCs) with non-lichen sequences and were classified as singletons or outlier VCs in network analysis (Supplementary Fig. S5). The remaining 335 contigs formed 133 VCs across the 11 metagenomes (Fig. 1d), with limited overlap to previously published sequences (i.e., only 28 VCs contained cyanolichen viral contigs and IMG/RefSeq sequences) (Supplementary Fig. S5) or among different cyanolichen samples (Supplementary Fig. S6). Similarly, we observed little functional overlap among our contigs (i.e., 96% of cyanolichen viral protein clusters (PCs) were singletons), consistent with high viral functional diversity [7]. Increasing sequencing depth would likely recover additional viruses and greater overlap with other ecosystems and among samples, but the lack of VC overlap also may reflect novel viral diversity and high geographic turnover of non-cyanobacterial lichen-associated bacterial communities [3] similar to turnover of marine viral communities according to host identity and physical or chemical properties of the environment [8, 18].

Consistent with the ability of phages in other ecosystems to encode host genes to drive host metabolism [8, 9], 550 of 21855 predicted ORFs had significant matches to metabolic KEGG HMM profiles (Fig. 2; Supplementary Table S4). Although putative AMGs represent a small fraction of total ORFs, they occurred on 19% of contigs (249 contigs with 1–2 AMGs per contig; see Supplementary Tables S4 and S5) and included diverse KEGG pathways such as amino acid, nucleotide, and carbohydrate metabolism (Fig. 2; Supplementary Tables S6 and S7). One of the most abundant AMGs in cyanolichens was the *rfb* operon (21 contigs from 8 metagenomes), which is involved in the production of lipopolysaccharides and extracellular polymeric substances (EPS) in gram-negative bacteria [19] (Supplementary Table S7). The high number of contigs carrying the complete operon is consistent with the potential ecological importance of EPS for lichen thalli formation, water retention, and microbial communication (reviewed by Spribille et al. [20]). Putative AMGs also included KEGG pathways for secondary metabolism, although contigs did not contain complete secondary metabolite gene clusters.

**Fig. 2:**
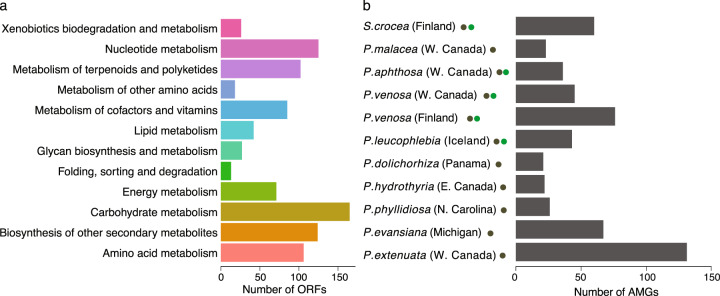
Cyanolichen viruses encode diverse auxiliary metabolic genes (AMGs) for amino acid, nucleotide, carbohydrate, and secondary metabolite metabolism. **a** Number of AMGs per KEGG pathway. In total, AMGs were found on 249 contigs, including 9 of the 27 circular viral genomes. Most putative cyanolichen viral contigs contained one or two ORFs with KEGG annotation (Supplementary Table S4). **b** Number of AMGs per cyanolichen sample.

In conclusion, numerous tools and approaches have been used to identify viruses in marine, human-associated, and soil metagenomes, yet viral detection remains challenging in complex and under-explored eukaryotic host-associated metagenomes. Here, we illustrate the diversity, novelty, and functional potential of viruses in cyanolichens and identify AMGs for metabolic pathways not previously described in viruses in other ecosystems (Supplementary Table S7). The diversity of viral AMGs in cyanolichens, including numerous AMGs for secondary metabolism, suggests viruses may modify interactions among complex microbial partners within lichens. Although viral novelty, microbial complexity, and inability to easily culture hosts in cyanolichen metagenomes limited our ability to link AMGs to specific hosts, future work will seek to decipher viral-host associations and the functional roles of AMGs in the lichen symbiosis using single viral particle sequencing.

## Supplementary information

Supplementary Information

Table S2-S5

## Data Availability

Data used in this study are available at the National Center for Biotechnology Information (NCBI) Sequence Read Archive (SRA) (see Supplementary Table S1 for accession numbers). Predicted viral reads and AMGs are available on figshare (10.6084/m9.figshare.c.5444376.v1). All code is available on GitHub (https://github.com/aponsero/) including the complete pipeline for (i) identifying viral contigs using VirSorter, Vibrant, and MARVEL (Viral_hunt_snakemake); (ii) validating that contigs are of viral origin (Viral_confirmation_snakemake); and (iii) code to run VirFinder in parallel on a High-Performance Computer (HPC) (VirFinder_parrallel_eval).
